# TNF rs1800629 Genotype and Motor Response to Intensive Rehabilitation in Parkinson’s Disease: An Exploratory Study

**DOI:** 10.3390/ijms27219491

**Published:** 2026-10-24

**Authors:** Cristina Agliardi, Franca Rosa Guerini, Elisabetta Bolognesi, Milena Zanzottera, Francesca Lea Saibene, Anna Salvatore, Pietro Arcuri, Anna Castagna, Davide Cattaneo, Thomas Bowman, Denise Anastasi, Jorge Navarro, Mario Meloni, Mario Clerici

**Affiliations:** 1 IRCCS Fondazione Don Carlo Gnocchi ETS, 20148 Milan, Italyebolognesi@dongnocchi.it (E.B.); fsaibene@dongnocchi.it (F.L.S.); asalvatore@dongnocchi.it (A.S.); tbowman@dongnocchi.it (T.B.);; 2 Pathophysiology and Transplantation Department, University of Milan, 20122 Milan, Italy; 3 Neurology Unit, Azienda Ospedaliero-Universitaria, 09042 Cagliari, Italy

**Keywords:** Parkinson’s disease, SNP, genetic polymorphism, TNF, rehabilitation, inflammation

## Abstract

Rehabilitation is a key component of Parkinson’s disease (PD) management, although individual responses differ substantially. Genetic factors involved in neuroplasticity and inflammation may contribute to this variability. We investigated whether selected polymorphisms in inflammation- and neuroplasticity-related genes influence motor outcomes following intensive multidisciplinary rehabilitation in patients with PD. Sixty-six patients with a diagnosis of idiopathic PD were consecutively enrolled and randomly allocated to an intensive multidisciplinary rehabilitation group (EXP-Group, *n* = 34) or a home-based stretching control group (CTRL-Group, n = 32) for six weeks. MDS-UPDRS III score was assessed at baseline (T0) and post-treatment (T1). SNPs in the *TNF*, *IL6*, *IL6R*, *IL1A*, *IL1B*, *IL10* and *BDNF* genes were genotyped. Possible associations between genotype, treatment group and MDS-UPDRS III score at T1 were analyzed using ANCOVA and linear mixed-effects models, adjusting for baseline MDS-UPDRS III score, age, sex, disease duration and levodopa equivalent daily dose (LEDD). A significant treatment group x *TNF rs1800629* genotype interaction was observed (*p* = 0.033). Among EXP-Group patients, *GG* homozygotes showed lower adjusted MDS-UPDRS III scores than did the *A*-allele carriers (29.10 vs. 36.11; *p* = 0.029). Longitudinal analyses revealed a significant time × treatment group × *TNF rs1800629* genotype interaction (*p* = 0.010), with *GG* homozygotes showing an approximately 10-point reduction in MDS-UPDRS III score (40.87 to 30.87; *p* < 0.001). These findings suggest that *TNF rs1800629* contributes to variability in response to intensive multidisciplinary rehabilitation in PD.

## 1. Introduction

Parkinson’s disease (PD) is a progressive neurodegenerative disorder characterized by motor and non-motor symptoms, for which rehabilitation represents a key component of multidisciplinary management. Multidisciplinary rehabilitation programs have been shown to improve symptoms of PD, including motor function, although the magnitude of individual response is highly variable [1].

Increasing evidence suggests that genetic factors contribute to inter-individual variability in response to rehabilitation across different diseases, including neurological disorders such as Alzheimer’s disease [2], stroke [3] and traumatic brain injury [4]. In particular, genetic variants affecting neuroplasticity, such as *BDNF* polymorphisms, as well as inflammatory pathways, may influence the biological mechanisms underlying recovery and adaptation to motor rehabilitation [5]. Neuroinflammation plays a pivotal role in the pathogenesis and progression of PD and is driven by microglial activation and the chronic release of inflammatory cytokines, including TNF-α, IL-1 and IL-6 [6,7,8]. Physical exercise and rehabilitation have been shown to promote neuroprotection and neuroplasticity, partly through the modulation of inflammatory pathways and the shift in the immune response toward an anti-inflammatory profile [9]. Therefore, functional single nucleotide polymorphisms (SNPs) in genes involved in inflammation and neuroplasticity may contribute to inter-individual differences in the response to rehabilitation. However, whether genetic variants influence the response to multidisciplinary rehabilitation in patients with PD remains unclear. 

In this study, we evaluated the potential association of selected SNPs in genes involved in neuroplasticity and inflammation (*BDNF rs6265 C/T*, *TNF rs361525 A/G* and *rs1800629 A/G*; *IL6 rs1800795 C/G*; *IL6R rs2228145 C/A*; *IL1A rs3783521 A/G*; *IL1B rs1143634 G/A*, *rs16944 G/A* and *rs1143627 G/A*; *IL10 rs1800896 T/C* and *rs1800872 T/G*) with motor outcomes in patients with PD undergoing an intensive multidisciplinary rehabilitation program [10]. The *BDNF rs6265* polymorphism is a widely studied SNP that results in a valine-to-methionine substitution and affects intracellular trafficking and the activity-dependent secretion of BDNF [11]. The *TNF* SNPs *rs361525* and *rs1800629* are both located in the promoter region and have been associated with changes in TNF-α expression, potentially influencing disease susceptibility and clinical outcomes [12,13,14]. The additional polymorphisms in the *IL6*, *IL6R*, *IL1A*, *IL1B* and *IL10* genes were selected because of their involvement in regulation of inflammatory signaling, as well as previous evidence linking them to functional recovery following rehabilitation in other pathologies [4,15,16,17]. 

We hypothesized that genetic variability in these pathways might contribute to inter-individual differences in rehabilitation outcomes, potentially through differences in the biological processes involved in inflammation and neuroplasticity. Specifically, we investigated whether the association between treatment group and post-treatment motor severity, assessed using the MDS-UPDRS Part III rating scale during the “ON” medication state, differed according to genotype.

## 2. Results

### 2.1. Baseline Characteristics

The baseline demographic and clinical characteristics of the study population are summarized in Table 1 (Table 1). PD patients were divided into two groups (EXP-Group and CTRL-Group). At baseline, the groups were comparable in terms of sex distribution, age at enrollment, disease duration, MDS-UPDRS-Part III score and levodopa equivalent daily dose (LEDD).

### 2.2. SNPs Genotypes

All investigated SNPs were in Hardy–Weinberg equilibrium (all *p* > 0.05). Genotypic and allelic distributions of all the studied polymorphisms are reported in Table 2.

### 2.3. TNF rs1800629 Genotype and Response to Intensive Multidisciplinary Rehabilitation

ANCOVA tests were performed to investigate the association between the treatment group and SNPs genotypes with MDS-UPDRS III score at T1, adjusting for MDS-UPDRS III score at T0, age, sex, disease duration and LEDD at T0. The overall model was proven to be statistically significant for *TNF rs1800629* SNP (F(8,57) = 17.41; *p* < 0.001; R^2^ = 0.710). MDS-UPDRS III score at T0 was strongly associated with MDS-UPDRS III score at T1 (F = 63.911; *p* < 0.001; partial η^2^ = 0.529). Importantly, a significant interaction between group and *TNF rs1800629* genotype was observed (F(1,57) = 4.80; *p* = 0.033; partial η^2^*p* = 0.078), indicating that the association between the *TNF rs1800629* genotype and MDS-UPDRS III at score T1 differed according to the treatment group. Notably, in the EXP-Group, *AG*+*AA* carriers showed significantly higher adjusted MDS-UPDRS III scores than those of the *GG* patients (36.11 vs. 29.10; mean difference = 7.01, 95% CI = 0.76 to 13.26; *p* = 0.029). Estimated marginal means of MDS-UPDRS III score at T1, according to group and *TNF rs1800629* genotype, and pairwise comparisons are presented in Table 3 (Table 3). In contrast to what was observed in the EXP-Group patients, in the CTRL-Group, no significant difference in adjusted MDS-UPDRS III scores was observed between *AG*+*AA* and *GG* carriers (35.01 vs. 38.02; mean difference = −3.00, 95% CI = −10.03 to 4.02; *p* = 0.396). When comparing the EXP- and CTRL- groups within each genotype, no significant difference was observed among *AG*+*AA* carriers (mean difference = −1.09, 95% CI −9.21 to 7.02; *p* = 0.789). Conversely, among *GG* patients, the MDS-UPDRS III score was significantly higher in the CTRL-Group than in the EXP-Group (38.02 vs. 29.10; mean difference = 8.92, 95% CI 4.83–13.02; *p* < 0.001). Between-group pairwise comparisons were adjusted using Bonferroni correction.

### 2.4. Longitudinal Analysis of MDS-UPDRS III Score

**Figure 1:**
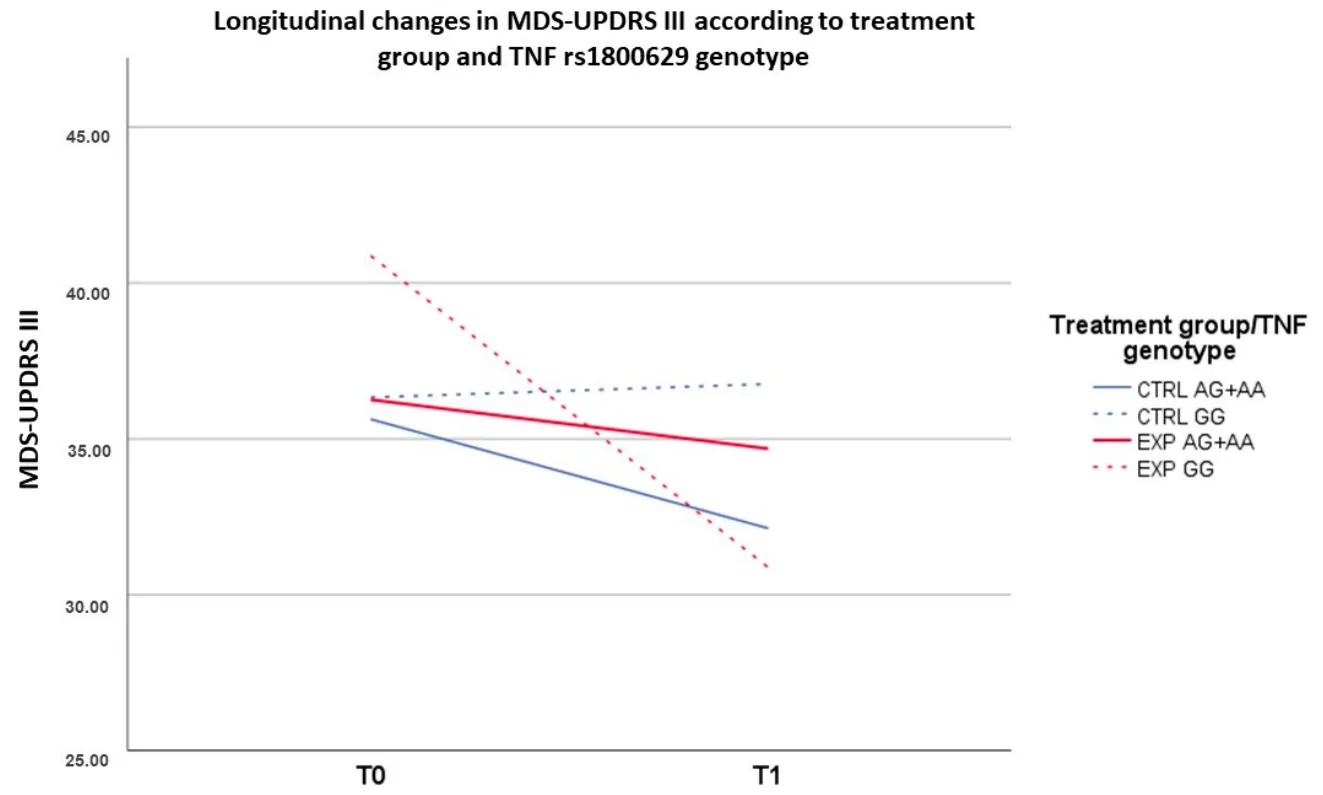
**Longitudinal changes in MDS-UPDRS III scores according to treatment group and *TNF rs1800629* genotype.** Estimated marginal means of MDS-UPDRS-III score at baseline (T0) and follow-up (T1) in the CTRL-Group and EXP-Group, stratified by *TNF rs1800629* genotype. The greatest longitudinal reduction in MDS-UPDRS III scores was observed in patients with the *GG* genotype in the EXP-Group, with adjusted scores decreasing from 40.87 at T0 to 30.87 at T1 (mean change = −10.00; *p* < 0.001). Lines represent estimated marginal means.

A linear mixed-effects model was subsequently created to evaluate longitudinal changes in the MDS-UPDRS III scale score between T0 and T1 and to determine whether these changes differed according to treatment group and *TNF rs1800629* genotype. A significant main effect of time was observed (F(1,62) = 10.063; *p* = 0.002). Importantly, a significant three-way interaction between time, treatment group and *TNF rs1800629* genotype was detected (F(1,62) = 7.155; *p* = 0.010), indicating that longitudinal changes in motor severity differed according to the combination of treatment group and genotype. Estimated marginal means showed that the most pronounced longitudinal reduction in motor severity occurred among *GG* homozygotes in the EXP-Group, whose adjusted scores decreased from 40.87 at T0 to 30.87 at T1 (mean change = −10.00; *p* < 0.001), whereas no significant within-group changes were observed in the other genotype-by-treatment subgroups (Figure 1). Pairwise comparisons were adjusted using Bonferroni correction. These findings further support a genotype-dependent response to intensive multidisciplinary rehabilitation.

### 2.5. Sensitivity Analysis Based on Changes in MDS-UPDRS III Score

As a sensitivity analysis, ΔMDS-UPDRS III score (T1−T0) was analyzed. The overall model was statistically significant (F(7,58) = 4.280; *p*< 0.001, η^2^*p* = 0.341). Consistent with the primary and longitudinal analyses, a significant treatment group x *TNF rs1800629* genotype interaction was observed (F(1,59) = 5.088; *p* = 0.028; η^2^*p* = 0.081). Thus, EXP-Group *GG* homozygotes showed a significantly greater reduction in MDS-UPDRS III score compared with that for *GG* homozygotes in the CTRL-group (adjusted mean difference = −9.99, 95% CI, −14.20 to −5.78; *p* < 0.001).

## 3. Discussion

In this exploratory study, we investigated whether polymorphisms in genes related to inflammation and neuroplasticity may contribute to inter-individual variability in the motor response to intensive multidisciplinary rehabilitation in patients with PD. Results showed the presence of a significant interaction between the treatment group (EXP-Group or CTRL-Group) and the *TNF rs1800629* genotype, with a more pronounced improvement in MDS-UPDRS III score observed among *GG* homozygotes receiving intensive multidisciplinary rehabilitation. In particular, rehabilitation resulted in an approximately 10-point reduction in adjusted MDS-UPDRS III scores in *GG* homozygotes in the EXP-Group. These findings suggest that the motor response to intensive rehabilitation is not uniform across patients and could be influenced by genetic variability in inflammatory pathways.

*TNF rs1800629* has been proposed as a functional promoter polymorphism, with the *A* allele having been associated with increased *TNF* transcriptional activity and consequently, with higher TNF-α production in some experimental systems [18,19]. In particular, the effect of *rs1800629* on *TNF* transcription has been suggested to vary across cell types, with the most pronounced effect observed in macrophages [20]. Moreover, the *A* allele has been strongly associated with the MHC haplotype HLA-A1, B8, DR3, which is itself linked to increased TNF production [21]. However, the functional consequences of this polymorphism remain controversial [22]. Therefore, the association observed in our study between the *GG* genotype and a greater improvement in motor outcomes following intensive multidisciplinary rehabilitation should not be interpreted as direct evidence of reduced TNF-α production. 

TNF-α is a key pro-inflammatory cytokine involved in the regulation of immune and inflammatory responses. Both systemic and brain inflammation have been documented in PD patients in the early stages of the disease [23]. Several studies have reported higher serum TNF-α levels in PD patients compared with those in healthy controls, and increased serum TNF-α concentrations have been associated with more advanced Hoehn and Yahr stages and higher MDS-UPDRS III scores [24]. Sustained neuroinflammation and prolonged TNF-α release may exert neurotoxic effects and have been implicated in the mechanisms underlying neurodegeneration [25]. Conversely, exercise-based rehabilitation may promote motor learning and functional adaptation through activity-dependent neuroplasticity and may also modulate inflammatory signaling [26]. These processes provide a plausible biological framework in which genetic variability in inflammatory pathways could contribute to inter-individual differences in rehabilitation outcome.

In this context, the greater improvement in MDS-UPDRS Part III scores observed among patients with the *GG* genotype receiving intensive multidisciplinary rehabilitation suggests that genetic variability in TNF-related pathways may contribute to differences in treatment responsiveness. Given the involvement of TNF-α in neuroinflammatory processes associated with PD, differences in the regulation of this pathway may influence the biological environment in which rehabilitation-induced motor learning and neural adaptation occur. In particular, a less pronounced pro-inflammatory response could potentially favor the functional adaptations elicited by intensive rehabilitation. Nevertheless, the functional effects of the *rs1800629* polymorphism are not fully established, and TNF-α concentrations were not measured in our study. Therefore, the observed genotype-dependent differences provide a basis for further investigation of the mechanisms linking TNF-related genetic variability to rehabilitation outcomes.

To the best of our knowledge, no previous studies have investigated the influence of *TNF*-related genetic variability on the response to rehabilitative interventions in PD. Interestingly, though, a small study in patients with subacute brain injury suggested that the *TNF rs1800629 GA* genotype may be associated with a poorer functional response to rehabilitation, as reflected by smaller improvements in the Barthel index and POMA scores [4].

Regarding our results, notably, when genotype was not taken into account, the time × group interaction did not reach statistical significance, whereas the three-way time × group × genotype interaction was significant. This finding suggests that the overall treatment effect may mask clinically relevant heterogeneity in treatment responses across genotypes. From a translational perspective, these preliminary results support further investigation of genetic factors as potential contributors to individualized rehabilitation response in PD. 

Limitations should be acknowledged. First, the sample size was relatively small, and no a priori sample size calculation was performed for this exploratory genetic sub-study. This limitation is particularly relevant after stratification according to genotype and treatment group. Furthermore, the low frequency of the *AA* genotype required its combination with *AG* carriers according to a dominant genetic model. The small sample size, particularly within genotype x treatment subgroups, may have reduced the statistical power and the precision of the effect estimates. Second, although analyses were adjusted for relevant demographic and clinical variables, residual confounding cannot be excluded. Third, circulating TNF-α concentration was not assessed; therefore, the proposed biological interpretation remains hypothetical. Fourth, information regarding the use of anti-inflammatory medications was not systematically collected, preventing us from assessing their potential influence on inflammatory pathways and rehabilitation outcomes. Future studies should incorporate detailed medication records alongside inflammatory biomarker measurements.

## 4. Materials and Methods

### 4.1. Study Population

The present study was a nested exploratory genetic sub-study of a randomized controlled trial (RCT) (NCT05452655) conducted at the Center for Diagnosis and Rehabilitation of Parkinson’s Disease and Parkinsonisms (DiaRiaPARK) and at the Laboratory of Molecular Medicine and Biotechnology (LAMMB) of the IRCCS Fondazione Don Carlo Gnocchi in Milan, Italy. The study was approved by the Ethics Committee of the institution (Protocol n° 11_16/04/2020). From a total of 72 PD patients with a diagnosis of idiopathic PD, 66 were eligible for this sub-study. They were consecutively enrolled and randomly allocated to two different groups: 34 to the “experimental group” (EXP-Group) and 32 to the “control group” (CTRL-Group). The EXP-Group underwent six consecutive weeks of outpatient intensive multidisciplinary rehabilitation treatment, five days per week, including motor, cognitive, and speech therapy (160–180 min/day, of which 80 min were dedicated to motor rehabilitation). Motor rehabilitation included aerobic training, task-oriented balance exercises, and individualized physiotherapy. The CTRL-Group performed home-based stretching and active mobilization exercises for 40 min/day, five days per week, for six weeks. Further details on the rehabilitation program and eligibility criteria are reported by Saibene et al. [10]. Demographic and clinical data taken into account in the present study include age, sex, disease duration (years), MDS-UPDRS-Part III score and levodopa equivalent daily dose (LEDD). Clinical evaluations were conducted at baseline (T0) and after treatment (T1). All procedures were conducted in accordance with the principles of the Declaration of Helsinki, and written informed consent was obtained from all participants prior to their inclusion in the study.

### 4.2. Blood Collection and DNA Extraction

Whole blood samples were collected in EDTA-Vacutainer tubes (Becton Dickinson Co., Rutherford, NJ, USA), and genomic DNA was extracted using the standard phenol/chloroform method. The DNA concentration and purity was determined by a spectrophotometer (SmartSpec Plus, Bio-Rad, Irvine, CA, USA). DNA samples were stored at −20 °C until use.

### 4.3. SNPs Genotyping

SNPs genotyping was performed by allelic discrimination real-time PCR with pre-designed TaqMan assays (Thermo Fisher Scientific, Waltham, MA, USA) (Table 4). The PCR protocol consisted of an initial hot start at 95 °C for 10 min, followed by 40 cycles of 15 s at 94 °C and 1 min at 60 °C. Fluorescence detection was performed at 60 °C. Reactions were carried out in 10 μL volumes using TaqMan Genotyping Master Mix (Thermo Fisher Scientific, Waltham, MA, USA) on 96-well plates with a CFX96 instrument (Bio-Rad, Hercules, CA, USA). Control samples representing all possible genotypes, along with a negative control, were included in each run. Genotype assignment was based on manual interpretation of the fluorescence discrimination results. All 66 participants were successfully assigned a genotype for each of the 11 investigated SNPs, corresponding to a genotype call rate of 100%. No samples were excluded because of unsuccessful genotype assignment, and no samples were repeated. Hardy–Weinberg equilibrium was assessed for all investigated polymorphisms as an additional quality-control measure.

### 4.4. Statistical Analysis

Statistical analyses were performed using IBM SPSS software (version 29.0.1.0, IBM, USA). Continuous variables were summarized as means and standard deviations (SDs), and categorical variables were summarized as frequencies and percentages. Hardy–Weinberg equilibrium was tested for each genetic SNPs distribution, applying Chi-square or Fisher’s exact tests, as appropriate. For the *TNF rs1800629* polymorphism, *AG* and *AA* genotypes were combined as *A* allele carriers (*AG*+*AA*) and compared with the *GG* genotype, according to a dominant genetic model. This approach was adopted because of the low frequency of the AA genotype, which limited the reliability of separate genotype-specific analyses. Chi-squared statistics were applied to 2XN tables, where appropriate, to compare sex distributions and genotypic and allelic distributions of the polymorphisms between the EXP-Group and CTRL-Group. Two-sided *p* values were considered significant at <0.05; Bonferroni correction was applied to post hoc pairwise comparisons to account for multiple comparisons within the corresponding analyses. An independent-sample t-test was applied to compare age at enrollment, disease duration, MDS-UPDRSIII scores and LEDD variables to test any difference between the EXP-Group and CTRL-Group at baseline (T0). 

The association between group, genotypes and motor severity measured by MDS UPDRS- III score was evaluated using a general linear model (ANCOVA), with MDS-UPDRS III score at T1 as the dependent variable. The model included the following variables: group, genotype, sex, age, LEDD at T0, disease duration and group × genotype interaction. MDS-UPDRS-III score at T0 was included as a covariate to account for baseline motor severity. Estimated marginal means were calculated for the group × genotype combinations, and pairwise comparisons were adjusted with Bonferroni correction. The assumptions of homogeneity of variance were assessed using Levene’s test. To investigate longitudinal changes in motor severity and determine whether the trajectory from baseline to post-treatment differed according to treatment group and genotype, a linear mixed-effects model was subsequently created. MDS-UPDRS-III score was entered as the dependent variable, with time (T0 and T1) specified as a within-subject factor and treatment group, genotype, and sex as between-subject factors. Age, disease duration, and LEDD at T0 were included as covariates. The model included the two-way interactions, time × genotype and group × genotype, as well as the three-way interaction, time × group × genotype, with the three-way interaction representing the primary effect of interest. Estimated marginal means and Bonferroni-adjusted pairwise comparisons were used to explore significant interactions. As a sensitivity analysis, change in motor severity was evaluated using the ΔMDS-UPDRS III score, calculated as the difference between the post-treatment and baseline scores (T1−T0). A univariate ANCOVA was performed, with the ΔMDS-UPDRS III score as the dependent variable; treatment group and genotype as fixed factors; their interaction as the main effect of interest; and age, sex, disease duration, and LEDD at T0 as covariates. Bonferroni-adjusted estimated marginal means and pairwise comparisons were used to characterize significant interactions.

## 5. Conclusions

In conclusion, our findings suggest that the *TNF rs1800629* genotype may contribute to the variability in motor response to intensive multidisciplinary rehabilitation in PD patients, with the greatest improvement observed among *GG* homozygotes. However, the limited sample size, the exploratory nature of the analyses and the absence of direct inflammatory biomarker measurements warrant cautious interpretation. Larger, adequately powered studies integrating genetic, inflammatory, and clinical measures are needed to determine whether TNF-related genetic variability could be a candidate biomarker in the context of personalized rehabilitation approaches in PD.

## Figures and Tables

**Table 1:** Baseline demographic and clinical characteristics of the study population.

Study Population Characteristics			
	EXP-Group	CTRL-Group	*p*
**N**	34	32	
**Sex M/F**	18/16	20/12	0.465
**Age at enrollment (years), mean ± SD**	70.00 ± 5.41	69.88 ± 7.23	0.937
**Disease duration T0** **(years), mean ± SD**	7.85 ± 5.69	7.42 ± 4.33	0.727
**MDS-UPDRS III T0** **mean ± SD**	40.76 ± 12.72	35.88 ± 12.75	0.124
**LEDD T0** **mean ± SD**	674.04 ± 342.99	608.06 ± 313.59	0.417

A Chi-square test was applied for the categorial variable: sex; an independent sample t-test was applied for the continuous variables: age at enrollment, disease duration at T0, MDS-UPDRS III score at T0 and LEDD at T0.

**Table 2:** Genotypic and allelic frequencies of SNPs in the study population.

***TNF rs361525***				
	**EXP-Group**	**CTRL-Group**	***p***	**H-W Equilibrium**
**Genotype**	**N (%)**	**N (%)**		
**AA**	0 (0.0)	0 (0.0)		
**AG**	2 (5.9)	2 (6.3)		
**GG**	32 (94.1)	30 (93.8)		
**Total**	34	32	0.670	0.99
**Allele**				
**A**	2 (2.9)	2 (3.1)		
**G**	66 (97.1)	62 (96.9)		
**Total**	68	64	0.954	
***TNF rs1800629***				
	**EXP-Group**	**CTRL-Group**	***p***	**H-W equilibrium**
**Genotype**	**N (%)**	**N (%)**		
**AA**	0 (0.0)	1 (3.1)		
**AG**	7 (20.6)	5 (15.6)		
**GG**	27 (79.4)	26 (81.3)		
**Total**	34	32	0.524	0.54
**Allele**				
**A**	7 (10.3)	7 (10.9)		
**G**	61 (89.7)	57 (89.1)		
**Total**	68	64	0.907	
***IL6 rs1800795***				
	**EXP-Group**	**CTRL-Group**	***p***	**H-W equilibrium**
**Genotype**	**N (%)**	**N (%)**		
**CC**	2 (5.9)	3 (9.4)		
**CG**	19 (55.9)	13 (40.6)		
**GG**	13 (38.2)	16 (50.0)		
**Total**	34	32	0.455	0.41
**Allele**				
**C**	23 (34.7)	19 (29.4)		
**G**	45 (65.3)	45 (70.6)		
**Total**	68	64	0.617	
***IL6R rs2228145***				
	**EXP-Group**	**CTRL-Group**	***p***	**H-W equilibrium**
**Genotype**	**N (%)**	**N (%)**		
**CC**	4 (11.8)	6 (18,8)		
**CA**	19 (55.9)	19 (59.4)		
**AA**	11 (32.4)	7 (21.9)	0.274	0.22
**Total**	34	32		
**Allele**				
**C**	27 (39.7)	31 (48.4)		
**A**	41 (60.3)	33 (51.6)		
**Total**	68	64	0.320	
***IL1A rs3783521***				
	**EXP-Group**	**CTRL-Group**	***p***	**H-W equilibrium**
**Genotype**	**N (%)**	**N (%)**		
**AA**	3 (8.8)	1 (3.1)		
**AG**	12 (35.3)	10 (31.3)		
**GG**	19 (55.9)	21 (65.6)		
**Total**	34	32	0.543	0.73
**Allele**				
**A**	18 (26.5)	12 (18.8)		
**G**	50 (73.5)	52 (81.2)		
**Total**	68	64	0.300	
***IL1B rs1143634***				
	**EXP-Group**	**CTRL-Group**	***p***	**H-W equilibrium**
**Genotype**	**N (%)**	**N (%)**		
**GG**	25 (73.5)	16 (50.0)		
**GA**	8 (23.5)	12 (37.5)		
**AA**	1 (2.9)	4 (12.5)		
**Total**	34	32	0.104	0.29
**Allele**				
**G**	58 (85.3)	44 (68.8)		
**A**	10 (14.7)	20 (31.2)		
**Total**	68	64	0.026	
***IL1B rs16944***				
	**EXP-Group**	**CTRL-Group**		
**Genotype**	**N (%)**	**N (%)**	***p***	**H-W equilibrium**
**GG**	17 (50.0)	17 (53.1)		
**GA**	14 (41.2)	12 (37.5)		
**AA**	3 (8.8)	3 (9.4)		
**Total**	34	32	0.954	0.77
**Allele**				
**G**	48 (70.6)	46 (71.9)		
**A**	20 (29.4)	18 (28.1)		
**Total**	68	64	0.874	
***IL1B rs1143627***				
	**EXP-Group**	**CTRL-Group**		
**Genotype**	**N (%)**	**N (%)**	***p***	**H-W equilibrium**
**GG**	3 (8.8)	3 (9.4)		
**GA**	15 (44.1)	12 (37.5)		
**AA**	16 (47.1)	17 (53.1)		
**Total**	34	32	0.859	0.99
**Allele**				
**G**	21 (30.9)	18 (28.1)		
**A**	47 (69.1)	46 (71.9)		
**Total**	68	64	0.734	
***IL10 rs1800896***				
	**EXP-Group**	**CTRL-Group**		
**Genotype**	**N (%)**	**N (%)**	***p***	**H-W equilibrium**
**TT**	15 (44.1)	14 (43.8)		
**TC**	16 (47.1)	14 (43.8)		
**CC**	3 (8.8)	4 (12.5)		
**Total**	34	32	0.882	0.99
**Allele**				
**T**	46 (67.6)	42 (65.6)		
**C**	22 (32.4)	22 (34.4)		
**Total**	68	64	0.808	
***IL10 rs1800872***				
	**EXP-Group**	**CTRL-Group**		
**Genotype**	**N (%)**	**N (%)**	***p***	**H-W equilibrium**
**TT**	3 (8.8)	3 (9.4)		
**TG**	10 (29.4)	12 (37.5)		
**GG**	21 (61.8)	17 (53.1)		
**Total**	34	32	0.762	0.33
**Allele**				
**T**	16 (23.5)	18 (28.1)		
**G**	52 (76.5)	46 (71.9)		
**Total**	68	64	0.554	
***BDNF rs6265***				
	**EXP-Group**	**CTRL-Group**		
**Genotype**	**N (%)**	**N (%)**	***p***	**H-W equilibrium**
**CC**	21 (61.8)	20 (62.5)		
**CT**	11 (32.4)	10 (31.3)		
**TT**	2 (5.9)	2 (6.3)		
**Total**	34	32	0.994	0.49
**Allele**				
**C**	53 (77.9)	50 (78.1)		
**T**	15 (22.1)	14 (21.9)		
**Total**	68	64	0.981	

H-W equilibrium = Hardy–Weinberg equilibrium.

**Table 3:** Adjusted MDS-UPDRS III scores at T1 according to treatment group and *TNF rs1800629* genotype.

**MSD UPDRS III at T1 Estimated Marginal Means**			
**Clinical Group**	***TNF rs1800629*** **Genotype**	**MSD UPDRS-III at T1 Adjusted Means ± SE**	**95% CI**
**CTRL-Group**	*AG*+*AA*	35.01 ± 3.09	28.83–41.20
**CTRL-Group**	*GG*	38.02 ± 1.48	35.05–40.99
**EXP-Group**	*AG*+*AA*	36.11 ± 2.76	30.58–41.64
**EXP-Group**	*GG*	29.10 ± 1.40	26.29–31.90
**MSD UPDRS III at T1 pairwise comparisons**			
**Pairwise comparison**	**Mean difference**	**95% CI**	**p_c_**
**CTRL-Group: *AG*+*AA* vs** **.** ***GG***	−3.00	−10.03 to 4.02	0.396
**EXP-Group: *AG*+*AA* vs** **.** ***GG***	7.01	0.76 to 13.26	0.029
***AG*** **+*AA*: CTRL-Group vs** **.** **EXP-Group**	−1.09	−9.205 to 7.023	0.789
***GG*** **: CTRL-Group vs** **.** **EXP-Group**	8.92	4.83 to 13.02	<0.001

Adjusted means were estimated from the ANCOVA model, including treatment group, *TNF rs1800629* genotype, sex, age at enrollment, MDS-UPDRS-III at T0, disease duration and the group x genotype interaction. Covariates were evaluated at their mean values. *AG*+*AA* indicates carriers of the *TNF rs1800629 A* allele under the dominant genetic model. Between-group pairwise comparisons were adjusted using Bonferroni correction (p_c_).

**Table 4:** List of selected SNPs with their rs identifiers and TaqMan assay IDs.

Gene	SNP	TaqMan Assay ID
***TNF***	*rs361525 A/G*	C___2215707_10
	*rs1800629 A/G*	C___7514879_10
***IL6***	*rs1800795 C/G*	C___1839697_20
***IL6R***	*rs2228145 C/A*	C__16170664_10
***IL1A***	*rs3783521 A/G*	C___1839900_10
***IL1B***	*rs1143634 G/A*	C___9546517_10
	*rs16944 G/A*	C___1839943_10
	*rs1143627 G/A*	C___1839944_10
***IL10***	*rs1800896 T/C*	C___1747360_10
	*rs1800872 T/G*	C___1747363_10
***BDNF***	*rs6265 C/T*	C__11592758_10

## Data Availability

The data presented in this study are available on request from the corresponding authors.

## Abbreviations

The following abbreviations are used in this manuscript:

ANCOVA	Analysis of covariance
BDNF	Brain derived neurotrophic factor
CI	Confidence interval
CTRL	Control
EXP	Experimental
F	F-statistics
LEDD	Levodopa equivalent daily dose
MDS-UPDRS III	Movement Disorder Society-Unified Parkinson’s Disease Rating Scale Part III
